# Metabolic signatures derived from whole-brain MR-spectroscopy identify early tumor progression in high-grade gliomas using machine learning

**DOI:** 10.1007/s11060-024-04812-1

**Published:** 2024-08-24

**Authors:** Cameron A. Rivera, Shovan Bhatia, Alexis A. Morell, Lekhaj C. Daggubati, Martin A. Merenzon, Sulaiman A. Sheriff, Evan Luther, Jay Chandar, Adam S. Levy, Ashley R. Metzler, Chandler N. Berke, Mohammed Goryawala, Eric A. Mellon, Rita G. Bhatia, Natalya Nagornaya, Gaurav Saigal, Macarena I de la Fuente, Ricardo J. Komotar, Michael E. Ivan, Ashish H. Shah

**Affiliations:** 1 Department of Neurosurgery, University of Miami Miller School of Medicine, Miami, FL USA; 2 Department of Neurosurgery, University of Pittsburgh School of Medicine, Pittsburgh, PA USA; 3 Department of Critical Care, University of Pittsburgh School of Medicine, Pittsburgh, PA USA; 4 Surgical Neuro-Oncology, District of Columbia, George Washington Medical Faculty Associates, Washington, USA; 5 Department of Neurosurgery, Yale University School of Medicine, New Haven, CT USA; 6 Department of Radiation Oncology, University of Miami Miller School of Medicine, Miami, FL USA; 7 Department of Radiology, University of Miami Miller School of Medicine, Miami, FL USA; 8 Department of Neurosurgery, Allegheny Health Network, Pittsburgh, PA USA; 9 Herbert Wertheim College of Medicine, Florida International University, Miami, FL USA; 10 Sylvester Comprehensive Cancer Center, University of Miami Miller School of Medicine, 1475 NW 12th Ave, Miami, FL USA; 11 Department of Neurology, University of Miami Miller School of Medicine, Miami, FL USA

**Keywords:** Machine learning, Glioblastoma, Whole-brain magnetic resonance spectroscopy (WB-MRS), Predictive models, Binary classification, Multiclass classifier

## Abstract

**Purpose:**

Recurrence for high-grade gliomas is inevitable despite maximal safe resection and adjuvant chemoradiation, and current imaging techniques fall short in predicting future progression. However, we introduce a novel whole-brain magnetic resonance spectroscopy (WB-MRS) protocol that delves into the intricacies of tumor microenvironments, offering a comprehensive understanding of glioma progression to inform expectant surgical and adjuvant intervention.

**Methods:**

We investigated five locoregional tumor metabolites in a post-treatment population and applied machine learning (ML) techniques to analyze key relationships within seven regions of interest: contralateral normal-appearing white matter (NAWM), fluid-attenuated inversion recovery (FLAIR), contrast-enhancing tumor at time of WB-MRS (Tumor), areas of future recurrence (AFR), whole-brain healthy (WBH), non-progressive FLAIR (NPF), and progressive FLAIR (PF). Five supervised ML classification models and a neural network were developed, optimized, trained, tested, and validated. Lastly, a web application was developed to host our novel calculator, the Miami Glioma Prediction Map (MGPM), for open-source interaction.

**Results:**

Sixteen patients with histopathological confirmation of high-grade glioma prior to WB-MRS were included in this study, totaling 118,922 whole-brain voxels. ML models successfully differentiated normal-appearing white matter from tumor and future progression. Notably, the highest performing ML model predicted glioma progression within fluid-attenuated inversion recovery (FLAIR) signal in the post-treatment setting (mean AUC = 0.86), with Cho/Cr as the most important feature.

**Conclusions:**

This study marks a significant milestone as the first of its kind to unveil radiographic occult glioma progression in post-treatment gliomas within 8 months of discovery. These findings underscore the utility of ML-based WB-MRS growth predictions, presenting a promising avenue for the guidance of early treatment decision-making. This research represents a crucial advancement in predicting the timing and location of glioblastoma recurrence, which can inform treatment decisions to improve patient outcomes.

**Supplementary Information:**

The online version contains supplementary material available at 10.1007/s11060-024-04812-1.

## Introduction

Recurrence for high-grade gliomas is inevitable despite maximal safe resection and adjuvant chemoradiation [1, 2]. High-grade gliomas are known to be highly infiltrative with fingerlike projections that may not entirely enhance under MRI T1-weighted-contrast-enhancement [3–5]. Although traditional MRI imaging modalities such as T2-weighted/fluid-attenuated inversion recovery sequences (FLAIR) are leveraged regularly in a clinician's armamentarium, these fall short in understanding the true extent of the non-contrast-enhancing tumor component. Whole-brain magnetic resonance spectroscopy (WB-MRS) offers the ability to detect subtle differences between key biomarkers that are present in the tumor microenvironment to better depict tumor extension [6–8].

Multiple metabolite changes have been identified with spectroscopy that mark the activity of mitotic cells and oncogenic drivers facilitating these changes. The interplay between key metabolites such as choline (Cho), creatine (Cr),* n*-acetyl-aspartate (NAA), glutamine-glutamate (Glx), and myo-inositol has been used to distinguish metabolic changes in high-grade glioma [7–14]. In tumor, Cho, a well-known biomarker for membrane proliferation and tumor growth, is typically increased, while NAA, a marker for healthy glial tissue, is typically decreased [10]. Additionally, Cr decreases [11] and Glx increases [12] in tumor due to increased metabolic demands, while myo-inositol decreases [13, 14] due to decreased concentration of osmoregulators resulting from breakdown of the blood–brain barrier. These five metabolic targets cumulatively provide a robust multiparametric snapshot of a voxel’s metabolic state.

**Fig. 1:**
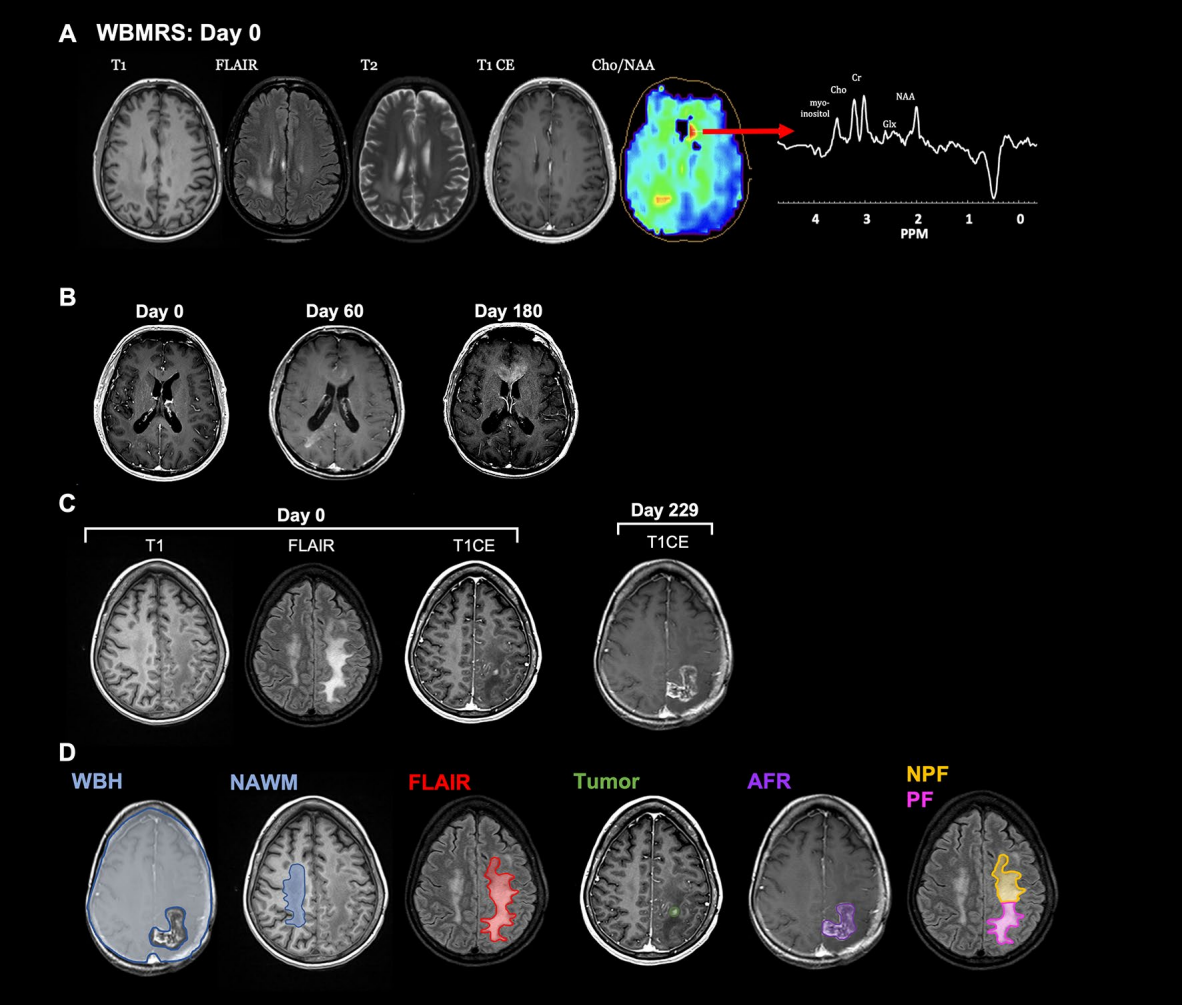
Proof-of-concept for Whole-Brain MR Spectroscopy (WB-MRS) in predicting glioma progression.** a** Evidence of WB-MRS as a predictor for distant recurrence and progression, through elevated Cho/NAA at Day 0 in the suspected area of tumor, as well as in the anterior corpus collosum. **b** Serial T1CE MRI on the same patient shows evident progression in the anterior corpus collosum by Day 180. **c** Sample case of a different patient with glioblastoma who previously underwent resection received WB-MRS on Day 0. Subsequent imaging at follow-up Day 229 shows evidence of progression. **d** Outline of regions of interest (ROIs) evaluated with WB-MRS. Seven ROIs are included. Whole brain healthy (WBH), Normal-appearing white matter (NAWM), fluid attenuation-inversion recovery (FLAIR), and Tumor are visualized at Day 0, while area of future recurrence (AFR), non-progressive FLAIR (NPF), and progressive FLAIR (PF) are identified at the time of progression

In the molecular era of gliomas, targeted treatment requires tools that promptly anticipates treatment-failure and disease progression. By noninvasively leveraging biomarkers for tissue proliferation, energy metabolism, and osmolarity, a more complete profile of future progression can serve this purpose. Cho/NAA ratios have slowly been integrated to assess abnormally proliferative tissue; however, neither whole-brain maps nor multiparametric assay have been included in current standard of care. Proof-of-concept evidence suggests whole-brain Cho/NAA maps (Fig. 1a) can be useful markers for future progression (Fig. 1b), prompting our hypothesis for the utility of additional WB-MRS metabolite maps in predicting recurrence.

Given the utility of machine learning (ML) in clinical modeling, supervised models may uncover key metabolic relationships beyond Cho/NAA values to predict future progression [15] by detecting spectrographic differences between key ROI’s. Here, we have piloted a novel WB-MRS protocol to investigate locoregional tumor metabolite signatures and implemented ML techniques to predict regions of tumor progression.

## Methods

### Study population

**Fig. 2:**
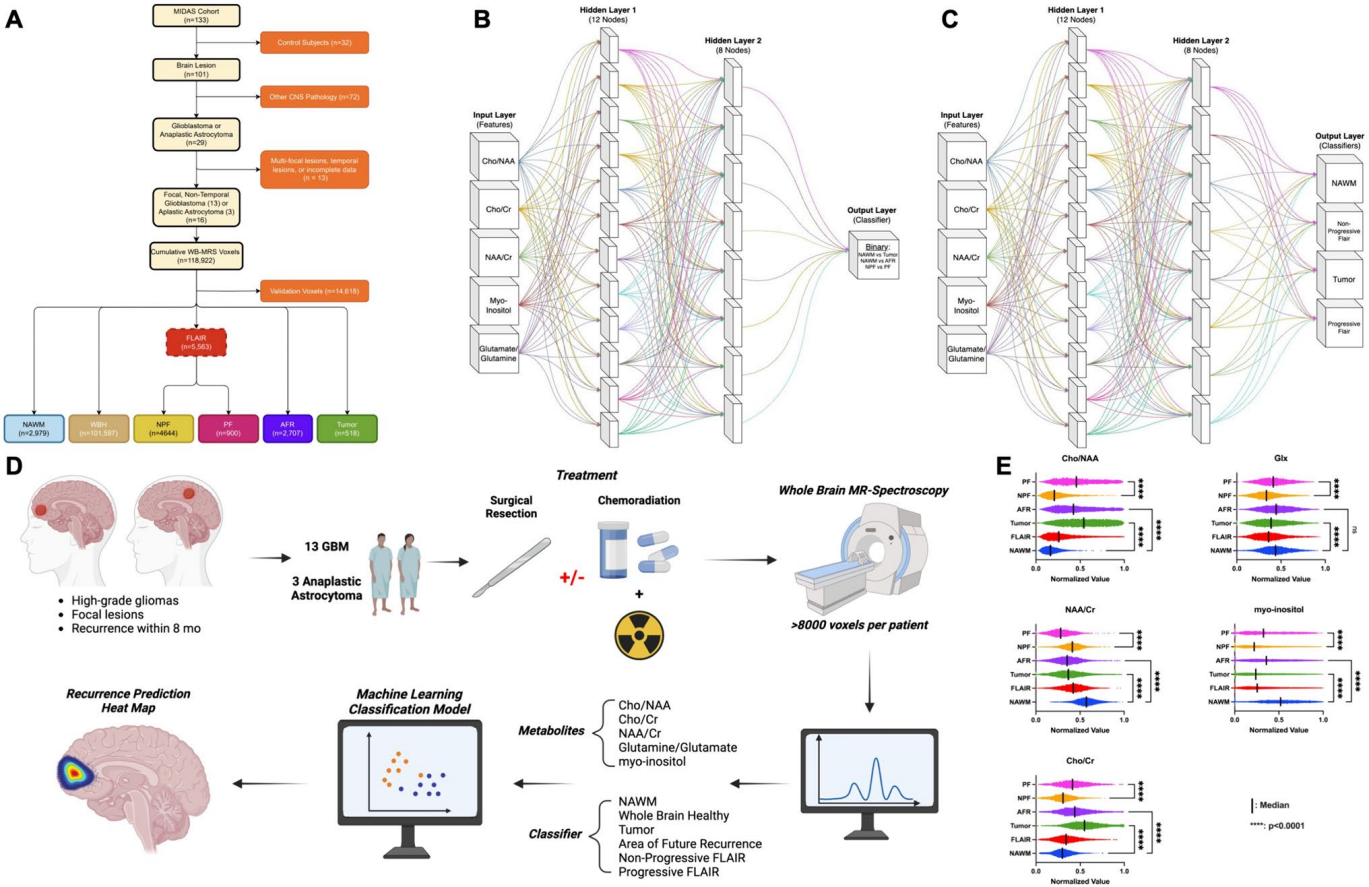
Study design. **a** Flow chart of patient and voxel inclusion and separation into six distinct regions of interest (ROI). The Metabolite Imaging and Data Analysis System (MIDAS), a software tool developed at the University of Miami, was used to evaluate WB-MRS. **b** Design structure of our deep learning artificial neural network (ANN) for binary classification. The ANN was constructed with Python’s keras & tensorflow libraries with 5 input nodes, 12 nodes in the first hidden layer, 8 layers in the second hidden layer, and 1 output layer. **c** Design structure of ANN for multi-class classification. All parameters matched the binary ANN except the output layer, which held four output nodes and a Softmax activation function. **d** Flow chart of the study design. Created with BioRender.com** e** Violin plots showing normalized values for key metabolites across different ROIs. NS, not significant

This prospective clinical study was conducted with approval from the institutional review board at the University of Miami. Study activities are outlined in Fig. 2a–e. In summary, 16 patients with surgical resection or chemoradiation for a high-grade glioma with histopathologic confirmation underwent WB-MRS and were found to show tumor progression within 8 months (Fig. 2d). Patient characteristics are summarized in Table 1.

**Table 1:** Patient demographic and clinical characteristics. Of 16 patients included in this study, 13 (81.3%) were diagnosed with GBM and 3 (18.7%) were diagnosed with anaplastic astrocytoma. IDH-1 and MGMT status were not reported for several patients; thus, demographic data was excluded from further ML analysis. The mean volume increase from baseline was 230.7% (SD: 143.8%)

Number of included patients		16
Age	Mean, years (SD)	58.0 (13.3)
Sex	Male, *N* (%)	9 (56.3%)
Primary pathology	GBM, *N* (%)	13 (81.3%)
	Anaplastic Astrocytoma, *N* (%)	3 (18.7%)
IDH1 status	Wild type, *N* (%)	7 (43.7%)
	Mutant, *N* (%)	2 (12.5%)
	Not reported, *N* (%)	7 (43.7%)
MGMT status	Methylated, *N* (%)	3 (18.7%)
	Unmethylated, *N* (%)	5 (31.3%)
	Not reported, *N* (%)	8 (50%)
Adjuvant therapy before WB-MRS	Yes, *N* (%)	9 (56.3%)
	No, *N* (%)	7 (43.7%)
Mean time after WB-MRS to progression, months (SD)		4.39 (1.89)
Volumetric analysis	Mean volume at MRS, cc (SD)	21.1 (18.0)
	Mean volume at progression, cc (SD)	32.6 (24.2)
	Mean % change, cc (SD)	230.7 (143.8)

### Imaging protocol

MRI data was acquired using a 3 Tesla Siemens Skyra MRI Scanner with a 20-channel head/neck coil. WB-MRS data was acquired using a 3D echo planar spectroscopic imaging (EPSI) sequence [16, 17]. Imaging parameters: TR/TE/TI = 1550/17.6/198 ms, excitation slab thickness = 140 mm, FA = 710, FOV = 280 × 280 × 180 mm, voxel resolution = 5.6 × 5.6 × 10, TA = 17 min. Pre-contrast T1, T2 and FLAIR images and a post-contrast T1 image were also acquired.

WB-MRS data processing was done using the MIDAS. The metabolite maps were obtained using the FITT module and metabolite ratios were derived from these maps. The NAWM maps were derived from the T1 image segmentation white matter tissue maps from the contralateral side of the tumor. The FLAIR and enhancing ROIs were created by segmentation of the FLAIR image and subtraction of the pre-contrast T1 from the post-contrast T1 image, respectively. Metabolite data was extracted using automated spectral analysis from the MIDAS software for Cho, NAA, Cr, Glx, and myo-inositol. This data was pre-processed using a built-in quality map and linewidth filter between 2 and 12 Hz.

Bone artifact around the temporal region has been shown to produce local magnetic field interference that decreases MR imaging quality [28], resulting in a MIDAS quality score below acceptable threshold. With multifocal lesions, the MIDAS ROI auto-segmentation capabilities struggled to classify concurrently enhancing lesions, thus precluding analysis of multifocal neoplasms.

### Voxel extraction & ROI’s

Seven key ROIs were generated from the Metabolic Imaging and Data Analysis System (MIDAS) [6, 16] and post-hoc analysis. Contralateral normal-appearing white matter (NAWM), FLAIR, and Tumor regions were automatically segmented from MIDAS. Under board-certified radiologist supervision, voxels that recurred at the first progressive MRI were hand-selected on the original Day 0 WB-MRS scan and labeled as area of future recurrence (AFR). Whole-Brain-Healthy (WBH) represented all voxels from the whole-brain scan that did not overlap with AFR. To further elucidate which voxels within the FLAIR became cancerous, the FLAIR was subdivided into areas of non-progressive FLAIR (NPF) and progressive FLAIR (PF) based on FLAIR overlap with the AFR region. ROIs are summarized in Fig. 1c–d. Unsupervised cluster analysis on these regions is outlined in Supplemental Methods 1.

### Machine learning classification models

#### Model architecture

Five supervised ML classification models were implemented in this analysis: Naive Bayes, Logistic Regression, Decision Tree, Random Forest, and Gradient Boosting (*scikit-learn*, Python 3.9.16). An additional deep-learning approach using an artificial neural network (ANN) was conceived to evaluate differences between traditional classifiers and deep learning (Fig. 2b–c). Hyperparameter tuning is outlined in Supplemental Methods 2. After hyperparameter tuning, all models were trained using a fivefold cross validation with a train/test split of 80/20% from 14 patients. Feature importance and model performance methodology are outlined in Supplemental Methods 3. Models predict voxel ROI classification into one of the two ROI’s used in the training phase.

#### Feature selection

Five metabolites were included for each voxel in all the datasets: Cho/NAA, Cho/Cr, NAA/Cr, myo-inositol, and Glx. Ratios were normalized to mean NAWM values, and smoothened maps of myo-inositol and Glx were used to minimize spectral variance. To correct for ROI volume variances, datasets were up-sampled and balanced using the Synthetic Minority Oversampling Technique (SMOTE) [17] such that an equal number of samples fall under each class.

#### Binary models

Four binary classification comparisons were made between key ROIs: NAWM vs. Tumor, NAWM vs. AFR, WBH vs. AFR, and NPF vs. PF. The final two comparisons were the outcomes of interest. The WBH vs. AFR served as a gross tool for differentiating glioma progression with whole-brain inputs, while the NPF vs. PF comparison was designed specifically for the FLAIR signal.

#### Multi-class model

Multi-class classification models were trained using NAWM, NPF, Tumor, and PF ROIs. Each voxel prediction was categorized into the ROI that yielded the highest predicted value.

#### Alternate training for binary machine-learning models

Cho/NAA is currently one of the strongest spectroscopic predictors for identifying tumor, and recent literature has shown a combination of Cho/NAA, NAA/Cr, and Cho/Cr may be sufficient in predicting tumor [8, 9]. Thus, we retrained the leading models with Cho/NAA alone and with the three ratios. Mean AUC and accuracy were recorded to measure the utility of these methods.

#### Cho/NAA threshold model

There is debate surrounding the thresholds of Cho/NAA necessary for identifying tumor. One hypothesized threshold of Cho/NAA for tumor within MR-spectroscopy is 2:1 [7]. Using the same fivefold cross validation testing sets as all binary paradigms, voxels were predicted as tumor/future progression if the Cho/NAA ratio was greater than 2:1. To find the optimal threshold, we incrementally changed the threshold until accuracy/AUC changed significantly [18].

### Validation

Two patients were randomized to our validation cohort. Whole-brain voxels were analyzed using the WBH vs. AFR binary ML model with the highest AUC. FLAIR voxels were also analyzed with the leading binary model. Procedure was repeated with the multi-class models. Each patient’s voxels were tested separately and then averaged.

Subsequently, a validation survey was created that included the patients’ brief histories and MRI scans for 17 suspected progression voxels chosen at random; these voxels were within the area of future recurrence but outside the current contrast-enhancing tumor boundaries. One voxel from the current contrast-enhancing region was added as a positive control to ensure survey integrity. Five neuroradiologists and neurosurgeons were prompted to predict if each voxel would progress within 6 months given the clinical and radiographic history. Results were compiled and compared along with our model’s predictions to ground truths. AUC and accuracy were primary outcome measures.

### Interactive application

To interface our model with external datasets, we developed a graphical user interface (GUI) application using *Shiny* architecture to predict a single voxel’s likelihood of future recurrence using our highest performing model retrained in RStudio.

## Results

### Individual voxels

A total of 16 patients with histopathological confirmation of high-grade glioma prior to WB-MRS were included in this study, totaling 118,922 whole-brain voxels. All patients had radiographic progression on an MRI within 8 months from the time of WB-MRS following the RANO [5] criteria (Table 1). Preliminary unsupervised hierarchical clustering analysis of NAWM, FLAIR, Tumor, and AFR voxels across all 5 metabolites correlated to ground-truth classifications (Supplementary Fig. 1). After pre-processing and up-sampling, ANOVA demonstrated significant differences between ROIs in three comparisons of interest: NAWM vs. Tumor, NAWM vs. AFR, NPF vs. PF (*p* < 0.0001) for all metabolites (Fig. 2e).

### Machine learning classification models

#### ML models differentiate normal-appearing white matter from tumor with strong AUCs (NAWM vs. tumor)

Six supervised ML classification models were trained and tested with NAWM and Tumor voxels across 14 patients as a positive control. Models performed with a highest mean AUC of 0.99. Feature importance was assessed for all classifiers, which consistently showed Cho/NAA as the most important feature. All models and their associated performance metrics are shown in Supplementary Tables 1 and 2 and Supplementary Fig. 2. To compare multiparametric ML models to a single Cho/NAA threshold definition of disease, Cho/NAA thresholds were tested at 0.1 intervals. The best-performing Cho/NAA threshold for differentiating Tumor from NAWM without machine-learning was 1.4 (AUC: 0.96) (Supplementary Fig. 9).

#### ML models differentiate normal-appearing white matter from areas of future recurrence (NAWM vs. AFR)

The next comparison expanded the Tumor region to the entire area of future recurrence (AFR). The NAWM vs. AFR classification models were similarly robust compared to the NAWM vs. Tumor models, with three models sharing the highest mean AUC (0.95). Similar to the NAWM vs. Tumor classifiers, Cho/NAA was the most important feature in the classical ML models, with NAA/Cr being the most important for the ANN. The other models and their associated performance metrics are shown in Supplementary Tables 1 and 2 and Supplementary Fig. 3. The most optimal Cho/NAA thresholds for differentiating these ROI’s were 1.3 and 1.4 (AUC: 0.90) (Supplementary Fig. 9).

#### ML models differentiate whole-brain healthy voxels from the area of future recurrence (WBH vs. AFR)

The third binary comparison was similar to NAWM vs. AFR but expanded the healthy voxels from contralateral NAWM to all whole-brain healthy voxels (WBH). Gradient Boosting showed the highest mean AUC (0.99). The most important feature for most models was Cho/Cr. The other models and their associated performance metrics are shown in Supplementary Tables 1 and 2 and Supplementary Fig. 4. The optimal Cho/NAA threshold was 1.6 (AUC: 0.82) (Supplementary Fig. 9).

#### ML models differentiate non-progressive FLAIR from progressive FLAIR (NPF vs. PF)

**Fig. 3:**
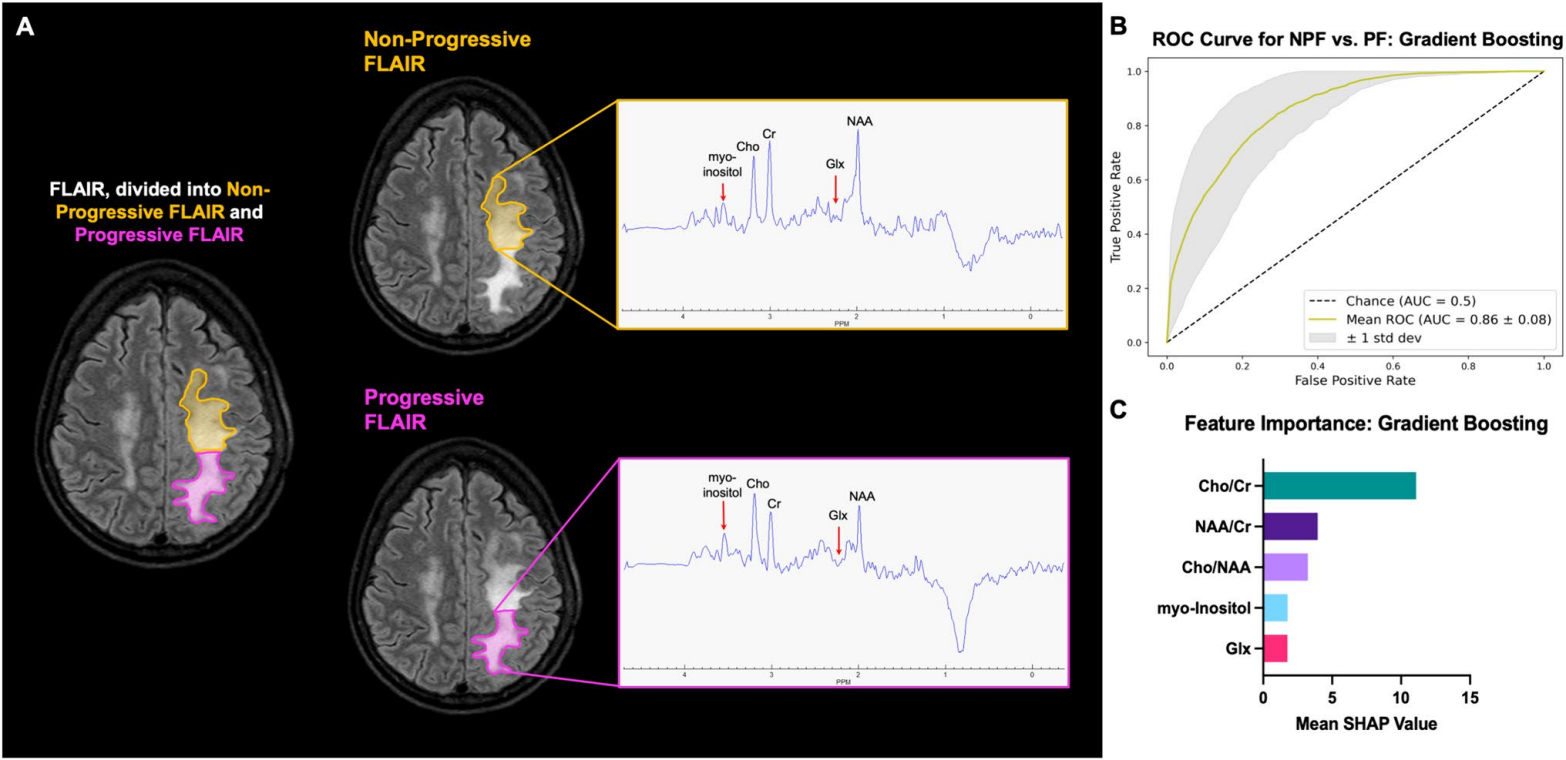
Binary classification of Non-Progressive FLAIR vs. Progressive FLAIR. **a** Delineation of the different regions and their associated spectra. **b** ROC for the highest performing Gradient Boosting model tested on FLAIR voxels. **c** Feature importances for the model, measured through Shapley (SHAP) values. This shows the multiparametric approach taken by the machine learning model, with each metabolite having an impact on the model’s prediction

The last binary comparison investigated in this study compared non-progressive and progressive FLAIR (NPF and PF), two regions which provided markedly different MR spectra between each other (Fig. 3a). Gradient Boosting had the highest mean AUC (0.86). Feature importance showed that Cho/NAA was the most important feature for all the models. The other models and their associated performance metrics are shown in Supplementary Tables 1 and 2 and Supplementary Fig. 5. The optimal Cho/NAA thresholds were 1.6, 1.7 and 1.8 (AUC: 0.75) (Supplementary Fig. 6). When the WBH vs. AFR Gradient Boosting model was applied to the FLAIR data set, the model also performed with a mean AUC of 0.86 but with marginally higher performance metrics compared to the NPF vs. PF Gradient Boosting model (Fig. 3b–c).

#### Multi-class classification model

As an extension to the NPF vs. PF modality, all six models were trained with NAWM, NPF, Tumor, and PF ROI’s using a multi-class modality, with results presented in Supplementary Tables 1 and 3 and Supplemental Fig. 7. Using One-vs-Rest (OvR) comparison, Gradient Boosting had the highest mean AUC (PF vs. Rest: 0.95).

### Validation

The WBH vs. AFR Gradient Boosting model tested on whole-brain voxels performed with a mean AUC of 0.863. Comparatively, a Cho/NAA threshold of 1.6 yielded a lower mean AUC of 0.749. We then applied the WBH vs. AFR Gradient Boosting model to FLAIR voxels, which performed with an average AUC of 0.727; a Cho/NAA threshold of 1.6 tested on FLAIR voxels resulted in an AUC of 0.597. To validate the five-metabolite approach, Gradient Boosting was retrained with relative ratios (Cho/NAA, Cho/Cr, NAA/Cr) and Cho/NAA-only. Across all paradigms, five-metabolite ML classifiers performed the best (Supplementary Tables 5 and 6). The Gradient Boosting PF vs. Rest multi-class model performed with an average AUC of 0.757. Validation AUC curves and confusion matrices are shown in Supplementary Figs. 8, 9 and 10. Lastly, a total of five physicians predicted future tumor progression from within FLAIR tissue with an average accuracy of 64.7% (AUC: 0.612), while the WBH vs. AFR Gradient Boosting model performed with an accuracy of 70.6% (AUC: 0.694). Cohen’s Kappa coefficient amongst responses was 0.297 (Supplementary Fig. 11).

### Sample case and prediction overlay of validation patients

**Fig. 4:**
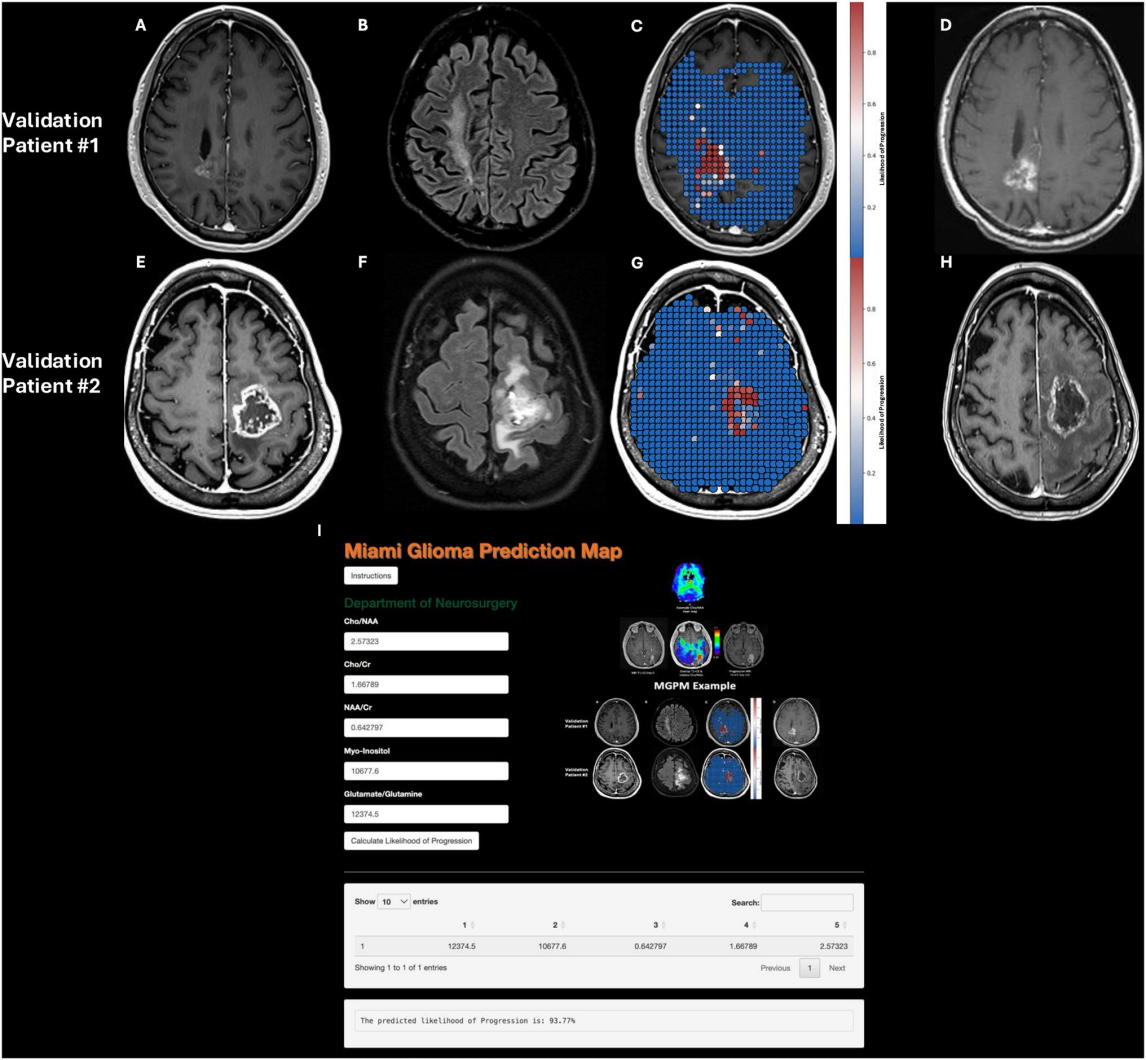
Sample case studies from the validation cohort. Axial imaging from Patient 1 showing **a** T1CE and **b** FLAIR MRI of stable disease post-operatively at time of WB-MRS (Day 0).** c** Indexed MRIs overlayed with prediction heatmap from the highest performing WB-MRS ML model are also included. **d** T1CE and FLAIR MRI 4 months later shows evidence of progressive disease.** e**–**h** Analogous imaging for Patient 2, who shows recurrence at 6 months.** i** Miami Glioma Prediction Map Interactive Web Application. Users can input single-voxel values for each metabolite which are processed to predict the likelihood of progression using the highest performing WB-MRS ML model

A patient in their 60’s who presented with confusion and vision loss underwent subtotal resection for GBM followed with concurrent chemoradiation. Serial imaging demonstrated stable contrast-enhancing disease (Fig. 4a) over the course of 6 months despite evidence of progressive, nonspecific FLAIR changes (Fig. 4b). WB-MRS at that time demonstrated marked locoregional differences in tumor-specific metabolites. Using predictions from the WBH vs. AFR Gradient Boosting classifier, a heatmap was generated of suspected tumor progression and overlaid onto the original MRI at the time of WB-MRS (Fig. 4c). Follow-up imaging over the next 5 months demonstrated progressive contrast-enhancing disease within the metabolically abnormal WB-MRS FLAIR region, suggesting the true extent of the lesion was radiographically occult (Fig. 4d).

A second patient in their 70’s presented for follow-up of known left frontal GBM. WB-MRS was conducted 1 month after sub-total resection (Fig. 4e-f), with model predictions for disease progression overlayed in Fig. 4g. Disease Progression at 6-month follow-up is shown in Fig. 4h.

### Interactive application

An interactive GUI application was developed to interface with the trained models, named the Miami Glioma Prediction Map (MGPM). The calculator predicts the likelihood of recurrence in single voxels using the WBH vs. AFR Gradient Boosting model (Fig. 4i). The application is linked here: https://cameron-rivera-development.shinyapps.io/UMiami_MGPM/. Due to the research nature of this model, the application does not provide clinical-grade prognostic information and should not be used in clinical-decision making; rather this model aims to invite interaction and collaboration within the scientific community as this technology is investigated.

## Discussion

The inevitable recurrence of high-grade gliomas sparks debate over the extent of clinical and surgical management at the time of diagnosis. Multimodal imaging is currently gold standard for diagnosing high-grade gliomas and is largely responsible for directing treatment decisions. However, the lack of unambiguous radiological features or biomarkers that accurately predict future progression demands a new approach; otherwise, the treating physician will always be making decisions after significant progression has already occurred. Previous investigation that has explored Cho/NAA and Cho/Cr metabolite ratios in classifying tumor recurrence has suggested possible merit to multiparametric spectrographic evaluation for tumor progression [19]. Based on the case study evidence presented in Fig. 1 coupled with unsupervised hierarchical clustering and differences within ROI metabolite levels (Fig. 2e), we hypothesized that ML models can extract complex metabolite relationships to predict progression up to 8 months in advance.

### Evaluating various machine learning algorithms

Across binary classifications, Gradient Boosting showed the highest AUCs, outperforming the more nuanced architecture of the ANN. While neural networks leverage sophisticated back-propagation and gradient descent algorithms to optimize their loss function, a less-robust tabular dataset, as we present here, may benefit more from a classical tree-based model, like Gradient Boosting, to avoid over-fitting [20]. Overall, our models’ abilities to utilize all 5 inputs shows even more convincingly that ML is a more robust statistical tool compared to traditional statistics in extracting complex relationships between variables.

### Differentiating areas of future recurrence

The NAWM vs. AFR binary classification models were developed to discern differences between healthy and imminently lesioned tissue. Although not entirely novel, this comparison still yielded interesting results that demonstrated how ML models are able to extract relationships between all 5 metabolites better than a Cho/NAA threshold. To the best of our knowledge, this initial model is the first modality of its kind to classify glioma progression prior to contrast-enhancement and served as a precursor to the WBH vs. AFR investigation.

While the successful NAWM vs. AFR binary classification was promising, the contralateral nature of the NAWM and the inclusion of Tumor voxels within the AFR region likely supported the ease of this classification. The WBH vs. AFR binary classification model aimed to consider each full scan in a simplified global approach and classify all voxels as healthy or unhealthy (defined as current tumor or future progression). The WBH voxels include both contralateral NAWM as well as voxels proximal to the lesion, allowing a holistic training set for non-progressive tissue. This unique facet may explain why most models relied on Cho/Cr heavily instead of Cho/NAA, but it remains unclear whether this preference for Cho/Cr can explain the high model performance. While the AFR region needed to be largely up-sampled, this comparison model seems to not only show strong model metrics but outperforms the NPF vs. PF models in differentiating FLAIR voxels.

### Differentiating tumor progression within FLAIR signal

In this study, we demonstrate markedly different MR spectra in two key regions within the FLAIR: NPF and PF. In current standard-of-care, successfully identifying non-enhancing tumors within the FLAIR is still a difficult task, even for experienced radiologists [21]. Through the comparison of NPF and PF, we were able to train our models to discern nuanced differences between these two, otherwise radiographically similar, regions.

As the bounds of safe supramaximal resection continue to be pushed in surgical neuro-oncology, understanding the true extent of the tumor within the FLAIR signal is necessary for guiding treatment decisions. Described in Shah et al. [2] and Di et al. [22], safe supramaximal resection of GBM lesions and surrounding FLAIR confers a survival benefit compared to gross total resection of the contrast-enhancing region alone. However, resecting non-contrast enhancing FLAIR can be limited by tumor location and eloquent tissue [23], and thus, delineation of progressive FLAIR voxels is of significance. When considering the short mean time to recurrence in patients with high-grade gliomas, there is an urgency for more proactive diagnostic evaluation to better inform surgical decision-making such that successful metabolic differentiation between NPF and PF could benefit clinicians facing the decision of when and where to resect.

Beyond high-grade pathologies, low-grade gliomas (LGG) may also benefit from a similar WB-MRS approach [24]. It is well known that LGGs are often characterized by their non-enhancing nature with high FLAIR sensitivity. WB-MRS may aid in evaluating the radiographic ambiguity of these slow-progressing pathologies, since the tradeoff between early intervention and operative morbidity remains unclear [25, 26].

### Cho/NAA threshold models

With previous research indicating Cho/NAA as a biomarker for tumor, we compared multi-parametric ML to a cutoff threshold of Cho/NAA for all binary comparisons. Notably, the Cho/NAA threshold was 1.6 for whole-brain monitoring, showing high accuracy and supporting previous research of this threshold for classifying high-grade gliomas [27]. Nevertheless, ML proved superior in all comparisons. One hypothesis for the poor validation of Cho/NAA is that the optimal cutoff for predicting progression may vary between patients, as evident in the validation phase. These results still provide support for Cho/NAA as a useful tool in predicting progression while multiparametric models are further investigated.

### Multi-class classification

A major drawback in training multiclass models is the physiological overlap in the underlying ROIs. The models most often confused PF with Tumor and NAWM with NPF, suggesting similar metabolic profiles in these regions (Supplementary Fig. 4). Given that neuroradiologists and neurosurgeons may treat Tumor and PF similarly in clinical practice, a combined “Pathologic” vs. “Non-Pathologic” outcome analysis like WBH vs. AFR may better influence the aggressiveness of supramarginal resection.

## Limitations

An important limitation of our study is the small sample size of 16 patients. Although all patients were classified as progression through RANO [5] criteria, only one patient had histopathological confirmation of progressive disease. Next, technical constraints to data acquisition excluded temporal and multifocal lesions from analysis. Regarding metabolites, current debate encircles water signal suppression on myo-inositol peaks as well as NAA overlap with Glx signal. Smoothened maps of these metabolites reduced noise, but the integrity of these two measurements should be further investigated. Furthermore, ROI sizes varied between patients; thus, the models weigh patients unequally. Additionally, up-sampling techniques such as SMOTE may add bias to our model. Validation was constricted to a limited sample size (*n* = 2). Further, our interactive application was not compatible with the Python model used to report results; while calculations are similar, we acknowledge subtle differences in the R and Python Gradient Boosting models.

## Conclusion

The future of non-invasive diagnostic imaging incorporating ML is poised to improve current treatment decision-making. This pilot study demonstrates the utility of a ML-based WB-MRS algorithm in predicting brain tumor progression and potentially guiding earlier treatment changes for optimal efficacy. Expanding the use of WB-MRS beyond research cohorts will improve our understanding of predictive models and the role for ML and radiomics in multiparametric imaging modalities.

## Supplementary Information

Below is the link to the electronic supplementary material.

Supplementary file1 (DOCX 8778 KB)

Supplementary file2 (DOCX 4479 KB)

## Data Availability

The data presented in this study are available from the corresponding author, upon reasonable request and institutional approval. Code Availability: All proprietary python and R code is available upon reasonable request to the corresponding author.
