# Supplementary material for: Metabolic signatures derived from whole-brain MR-spectroscopy identify early tumor progression in high-grade gliomas using machine learning
Source: J Neurooncol. 2024 Aug 24;170(3):579–89. doi: 10.1007/s11060-024-04812-1 (PMC11614968; doi:10.1007/s11060-024-04812-1)
Supplement: Supplementary file 1 — Supplementary file1 (DOCX 8778 KB) [file 11060_2024_4812_MOESM1_ESM.docx]

|  | | **Accuracy** | **Precision** | **Specificity** | **Sensitivity** | **F1-score** |
| --- | --- | --- | --- | --- | --- | --- |
| **Naïve Bayes** | |  |  |  |  |  |
| Binary | NAWM vs. Tumor | 0.932 | 0.986 | 0.986 | 0.876 | 0.928 |
|  | NAWM vs. AFR | 0.843 | 0.984 | 0.984 | 0.698 | 0.813 |
|  | WBH vs AFR | 0.720 | 0.983 | 0.903 | 0.494 | 0.638 |
|  | NPF vs. PF | 0.629 | 0.766 | 0.766 | 0.443 | 0.550 |
| Multiclass | NAWM vs. Rest | 0.799 | 0.720 | 0.720 | 0.331 | 0.443 |
|  | NPF vs. Rest | 0.733 | 0.059 | 0.059 | 0.012 | 0.018 |
|  | Tumor vs. Rest | 0.755 | 0.742 | 0.742 | 0.057 | 0.103 |
|  | PF vs. Rest | 0.754 | 0.574 | 0.574 | 0.146 | 0.229 |
| **Logistic Regression** | |  |  |  |  |  |
| Binary | NAWM vs. Tumor | 0.969 | 0.973 | 0.973 | 0.967 | 0.970 |
|  | NAWM vs. AFR | 0.829 | 0.887 | 0.887 | 0.798 | 0.821 |
|  | WBH vs AFR | 0.761 | 0.762 | 0.762 | 0.773 | 0.766 |
|  | NPF vs. PF | 0.594 | 0.594 | 0.594 | 0.685 | 0.633 |
| Multiclass | NAWM vs. Rest | 0.850 | 0.766 | 0.766 | 0.599 | 0.662 |
|  | NPF vs. Rest | 0.750 | 0.000 | 0.000 | 0.000 | 0.000 |
|  | Tumor vs. Rest | 0.783 | 0.615 | 0.615 | 0.394 | 0.473 |
|  | PF vs. Rest | 0.754 | 0.571 | 0.571 | 0.170 | 0.252 |
| **ANN** | |  |  |  |  |  |
| Binary | NAWM vs. Tumor | 0.979 | 0.979 | 0.979 | 0.979 | 0.979 |
|  | NAWM vs. AFR | 0.895 | 0.907 | 0.907 | 0.897 | 0.896 |
|  | WBH vs AFR | 0.835 | 0.831 | 0.831 | 0.842 | 0.837 |
|  | NPF vs. PF | 0.700 | 0.685 | 0.685 | 0.773 | 0.723 |
| Multiclass | NAWM vs. Rest | 0.899 | 0.805 | 0.805 | 0.798 | 0.793 |
|  | NPF vs. Rest | 0.763 | 0.543 | 0.543 | 0.316 | 0.396 |
|  | Tumor vs. Rest | 0.841 | 0.710 | 0.710 | 0.630 | 0.664 |
|  | PF vs. Rest | 0.802 | 0.709 | 0.709 | 0.439 | 0.529 |
| **Decision Tree** | |  |  |  |  |  |
| Binary | NAWM vs. Tumor | 0.974 | 0.969 | 0.969 | 0.981 | 0.975 |
|  | NAWM vs. AFR | 0.905 | 0.929 | 0.929 | 0.887 | 0.905 |
|  | WBH vs AFR | 0.914 | 0.899 | 0.899 | 0.932 | 0.915 |
|  | NPF vs. PF | 0.728 | 0.685 | 0.685 | 0.600 | 0.761 |
| Multiclass | NAWM vs. Rest | 0.905 | 0.792 | 0.792 | 0.846 | 0.816 |
|  | NPF vs. Rest | 0.767 | 0.602 | 0.602 | 0.235 | 0.332 |
|  | Tumor vs. Rest | 0.799 | 0.661 | 0.661 | 0.481 | 0.529 |
|  | PF vs. Rest | 0.776 | 0.672 | 0.672 | 0.276 | 0.383 |
| **Random Forest** | |  |  |  |  |  |
| Binary | NAWM vs. Tumor | 0.984 | 0.980 | 0.980 | 0.988 | 0.984 |
|  | NAWM vs. AFR | 0.897 | 0.905 | 0.905 | 0.895 | 0.896 |
|  | WBH vs AFR | 0.910 | 0.901 | 0.901 | 0.923 | 0.911 |
|  | NPF vs. PF | 0.773 | 0.728 | 0.728 | 0.887 | 0.798 |
| Multiclass | NAWM vs. Rest | 0.913 | 0.812 | 0.812 | 0.857 | 0.829 |
|  | NPF vs. Rest | 0.795 | 0.681 | 0.681 | 0.363 | 0.466 |
|  | Tumor vs. Rest | 0.938 | 0.880 | 0.880 | 0.870 | 0.875 |
|  | PF vs. Rest | 0.889 | 0.782 | 0.782 | 0.783 | 0.781 |
| **Gradient Boosting** | |  |  |  |  |  |
| Binary | NAWM vs. Tumor | 0.983 | 0.975 | 0.975 | 0.991 | 0.983 |
|  | NAWM vs. AFR | 0.887 | 0.890 | 0.890 | 0.894 | 0.888 |
|  | WBH vs AFR | 0.958 | 0.934 | 0.934 | 0.985 | 0.959 |
|  | *WBH vs AFR within FLAIR test voxels | 0.793 | 0.744 | 0.744 | 0.910 | 0.817 |
|  | NPF vs. PF | 0.791 | 0.741 | 0.741 | 0.912 | 0.816 |
| Multiclass | NAWM vs. Rest | 0.913 | 0.793 | 0.793 | 0.887 | 0.834 |
|  | NPF vs. Rest | 0.807 | 0.685 | 0.685 | 0.433 | 0.521 |
|  | Tumor vs. Rest | 0.941 | 0.856 | 0.856 | 0.923 | 0.888 |
|  | PF vs. Rest | 0.896 | 0.760 | 0.760 | 0.866 | 0.808 |

**Supplementary Table 1.** Performance metrics of ML models for binary and multi-class classifications. Highest performance metric is bolded in each category.

|  | **Mean AUC (± 1 SD)** |
| --- | --- |
| **Naïve Bayes** | |
| NAWM vs. Tumor | 0.99 ± 0.01 |
| NAWM vs. AFR | 0.95 ± 0.02 |
| WBH vs AFR | 0.87 ± 0.02 |
| NPF vs. PF | 0.74 ± 0.14 |
| **Logistic Regression** | |
| NAWM vs. Tumor | 0.99 ± 0.01 |
| NAWM vs. AFR | 0.92 ± 0.05 |
| WBH vs AFR | 0.91 ± 0.02 |
| NPF vs. PF | 0.63 ± 0.13 |
| **ANN** | |
| NAWM vs. Tumor | 0.98 ± 0.02 |
| NAWM vs. AFR | 0.90 ± 0.04 |
| WBH vs AFR | 0.84 ± 0.02 |
| NPF vs. PF | 0.70 ± 0.06 |
| **Decision Tree** | |
| NAWM vs. Tumor | 0.99 ± 0.01 |
| NAWM vs. AFR | 0.94 ± 0.04 |
| WBH vs AFR | 0.91 ± 0.01 |
| NPF vs. PF | 0.76 ± 0.09 |
| **Random Forest** | |
| NAWM vs. Tumor | 0.99 ± 0.00 |
| NAWM vs. AFR | 0.95 ± 0.03 |
| WBH vs AFR | 0.93 ± 0.01 |
| NPF vs. PF | 0.84 ± 0.08 |
| **Gradient Boosting** | |
| NAWM vs. Tumor | 0.99 ± 0.00 |
| NAWM vs. AFR | 0.95 ± 0.03 |
| WBH vs AFR | 0.99 ± 0.01 |
| *WBH vs AFR within FLAIR test voxels | 0.86 ± 0.08 |
| NPF vs. PF | 0.70 ± 0.10 |

**Supplementary Table 2.** Binary Classification ML Models and its associated AUCs +/- 1 SD. Includes all 6 machine-learning models and all 4 binary paradigms.

|  | ***Mean AUC*** |
| --- | --- |
| **Naïve Bayes** | |
| NAWM vs. Rest | 0.83 ± 0.08 |
| NPF vs. Rest | 0.68 ± 0.19 |
| Tumor vs. Rest | 0.71 ± 0.16 |
| PF vs. Rest | 0.70 ± 0.15 |
| **Logistic Regression** | |
| NAWM vs. Rest | 0.92 ± 0.03 |
| NPF vs. Rest | 0.76 ± 0.17 |
| Tumor vs. Rest | 0.78 ± 0.14 |
| PF vs. Rest | 0.77 ± 0.12 |
| **ANN** | |
| NAWM vs. Rest | 0.96 ± 0.01 |
| NPF vs. Rest | 0.84 ± 0.13 |
| Tumor vs. Rest | 0.86 ± 0.11 |
| PF vs. Rest | 0.86 ± 0.10 |
| **Decision Tree** | |
| NAWM vs. Rest | 0.95 ± 0.01 |
| NPF vs. Rest | 0.81 ± 0.16 |
| Tumor vs. Rest | 0.83 ± 0.18 |
| PF vs. Rest | 0.82 ± 0.12 |
| **Random Forest** | |
| NAWM vs. Rest | 0.97 ± 0.01 |
| NPF vs. Rest | 0.87 ± 0.11 |
| Tumor vs. Rest | 0.90 ± 0.11 |
| PF vs. Rest | 0.91± 0.09 |
| **Gradient Boosting** | |
| NAWM vs. Rest | 0.96 ± 0.01 |
| NPF vs. Rest | 0.87 ± 0.10 |
| Tumor vs. Rest | 0.91 ± 0.10 |
| PF vs Rest | 0.95 ± 0.02 |

**Supplementary Table 3.** Multi-class Classification ML Models observing the One vs. Rest technique and its associated AUCs

| **Naïve Bayes** | | **Hyperparameters** |
| --- | --- | --- |
| Binary | NAWM vs. Tumor | GaussianNB() |
|  | NAWM vs. AFR | GaussianNB() |
|  | WBH vs AFR | GaussianNB() |
|  | NPF vs. PF | GaussianNB() |
| Multiclass | NAWM vs. Rest | (var_smoothing=8.111308307896873e-08) |
|  | NPF vs. Rest |  |
|  | Tumor vs. Rest |  |
|  | PF vs. Rest |  |
| **Logistic Regression** | |  |
| Binary | NAWM vs. Tumor | LogisticRegression() |
|  | NAWM vs. AFR | LogisticRegression() |
|  | WBH vs AFR | (penalty='none', solver='newton-cg') |
|  | NPF vs. PF | LogisticRegression() |
| Multiclass | NAWM vs. Rest | (C=0.08858667904100823, multi_class = 'multinomial') |
|  | NPF vs. Rest |  |
|  | Tumor vs. Rest |  |
|  | PF vs. Rest |  |
| **ANN** | |  |
| Binary | NAWM vs. Tumor | (epochs=50, batch_size=10) |
|  | NAWM vs. AFR | (epochs=50, batch_size=10) |
|  | WBH vs AFR | (epochs=50, batch_size=10) |
|  | NPF vs. PF | (epochs=50, batch_size=10) |
| Multiclass | NAWM vs. Rest | (epochs=50, batch_size=10) |
|  | NPF vs. Rest |  |
|  | Tumor vs. Rest |  |
|  | PF vs. Rest |  |
| **Decision Tree** | |  |
| Binary | NAWM vs. Tumor | (max_depth=5, max_features=6, min_samples_leaf=8) |
|  | NAWM vs. AFR | (criterion='entropy', max_depth=3, max_features=5, min_samples_leaf=2) |
|  | WBH vs AFR | (max_features=8) |
|  | NPF vs. PF | (criterion='entropy', max_depth=3, max_features=6, min_samples_leaf=6) |
| Multiclass | NAWM vs. Rest | (criterion='entropy', max_depth=5, max_features=5, min_samples_leaf=8) |
|  | NPF vs. Rest |  |
|  | Tumor vs. Rest |  |
|  | PF vs. Rest |  |
| **Random Forest** | |  |
| Binary | NAWM vs. Tumor | (bootstrap=False, max_depth=80, max_features='auto',min_samples_split=10, n_estimators=1000) |
|  | NAWM vs. AFR | n_estimators = 100, oob_score = True) |
|  | WBH vs AFR | (max_depth=50, max_features='auto', bootstrap=False, min_samples_leaf=2, n_estimators=2000) |
|  | NPF vs. PF | (bootstrap=False, max_depth=60, max_features='auto',min_samples_leaf=2, n_estimators=600) |
| Multiclass | NAWM vs. Rest | (bootstrap=False, max_depth=50, max_features='auto', min_samples_leaf=2, n_estimators=2000) |
|  | NPF vs. Rest |  |
|  | Tumor vs. Rest |  |
|  | PF vs. Rest |  |
| **Gradient Boosting** | |  |
| Binary | NAWM vs. Tumor | (max_depth=5, min_samples_leaf=2, min_samples_split=4,n_estimators=500) |
|  | NAWM vs. AFR | (max_depth=5, max_features='auto', min_samples_leaf=6) |
|  | WBH vs AFR | (max_depth=15, max_features='auto', min_samples_leaf=2, n_estimators=1300) |
|  | NPF vs. PF | (max_depth=15, max_features='auto', min_samples_leaf=2, min_samples_split=10, n_estimators=1100) |
| Multiclass | NAWM vs Rest | (max_depth=15, max_features='sqrt',min_samples_leaf=2, n_estimators=1500) |
|  | NPF vs Rest |  |
|  | Tumor vs Rest |  |
|  | PF vs Rest |  |

**Supplementary Table 4.** Hyperparameters used for binary and multiclass ML classification models.

| **Gradient Boosting** | AUC: Cho/NAA Only | AUC: Relative Ratios Only |
| --- | --- | --- |
| NAWM vs Tumor | 0.97 ± 0.00 | 0.99 ± 0.00 |
| NAWM vs AFR | 0.94 ± 0.04 | 0.95 ± 0.03 |
| WBH vs AFR | 0.88 ± 0.01 | 0.96 ± 0.01 |
| NPF vs PF | 0.79 ± 0.03 | 0.86 ± 0.04 |

**Supplementary Table 5.** Validation of multiparametric binary ML. Gradient Boosting models were retrained with Cho/NAA only and again with only relative ratios (Cho/NAA, Cho/CR, NAA/CR; no myo-inositol or Glx). 5-fold cross-validated mean AUC with standard deviation were recorded. All mean AUC were lower than 5-fold cross-validated AUC from Gradient Boosting models trained on all 5 metabolites.

|  | **Accuracy** | **Precision** | **Specificity** | **Sensitivity** | **F1-Score** |
| --- | --- | --- | --- | --- | --- |
| **Cho/NAA Only** |  |  |  |  |  |
| *NAWM vs. Tumor* | 0.930 | 0.931 | 0.931 | 0.928 | 0.930 |
| *NAWM vs. AFR* | 0.894 | 0.934 | 0.934 | 0.849 | 0.888 |
| *WBH vs AFR* | 0.802 | 0.802 | 0.802 | 0.804 | 0.804 |
| *NPF vs. PF* | 0.704 | 0.699 | 0.699 | 0.720 | 0.709 |
| **Ratios Only** |  |  |  |  |  |
| *NAWM vs. Tumor* | 0.962 | 0.966 | 0.966 | 0.957 | 0.962 |
| *NAWM vs. AFR* | 0.904 | 0.932 | 0.932 | 0.874 | 0.900 |
| *WBH vs AFR* | 0.905 | 0.888 | 0.888 | 0.928 | 0.908 |
| *NPF vs. PF* | 0.788 | 0.767 | 0.767 | 0.834 | 0.798 |

**Supplementary Table 6**. Performance metrics for validation Gradient Boosting Cho/NAA only and ratios only models. Results are averaged across 5-fold cross-validation.


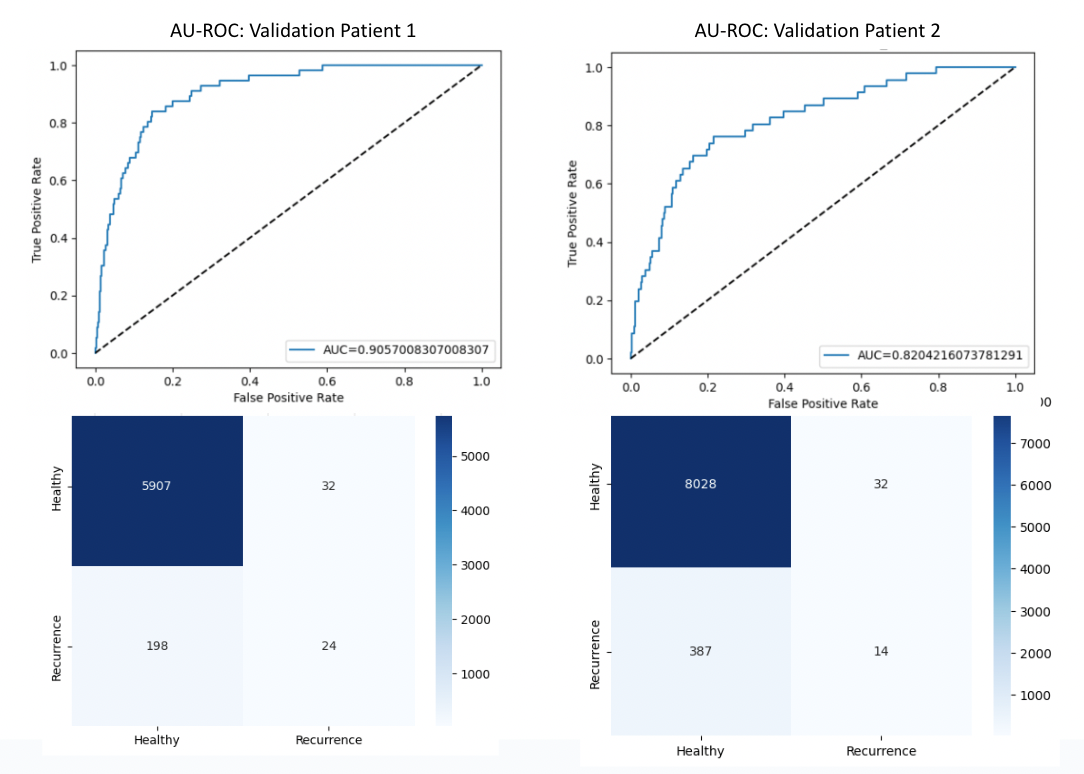


**Supplemental Figure 1:** Validation ROC and confusion matrices for Gradient Boosting trained and tested with WBH vs AFR voxels. Y-axis represents machine-learning predictions. X-axis represents supervised classifiers.


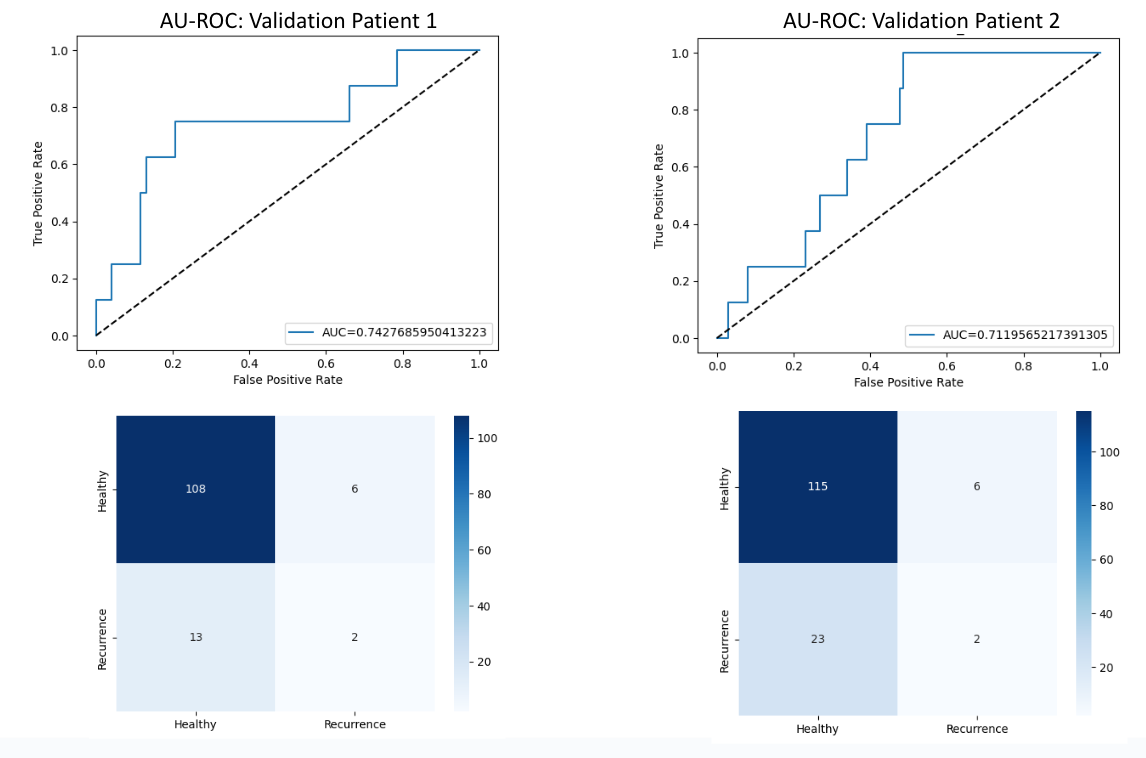


**Supplemental Figure 2:** Validation ROC and confusion matrices for Gradient Boosting trained with WBH vs AFR voxels and tested with NPF vs PF voxels. Confusion matrix y-axis represents machine-learning predictions; x-axis represents supervised classifiers.

`
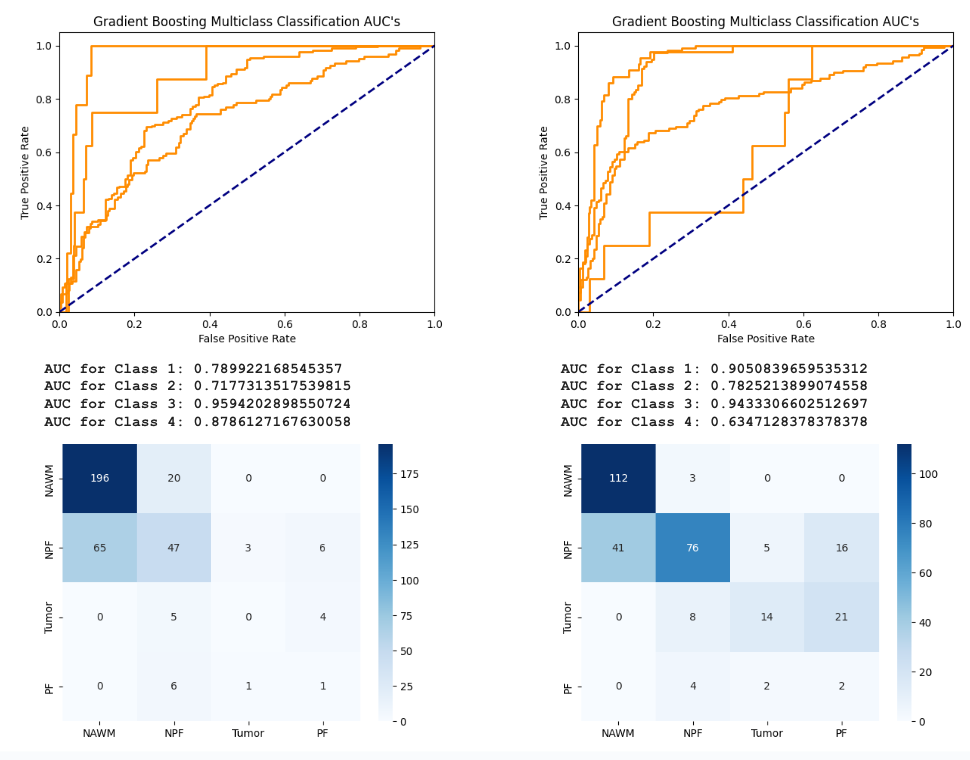


**Supplemental Figure 3:** Validation ROC’s and confusion matrices for Gradient Boosting Multi-Class. Results displayed per patient. Confusion matrix y-axis represents machine-learning predictions. X-axis represents supervised classifiers. Class 1: NAWM vs Rest; Class 2: NPF vs Rest; Class 3: Tumor vs Rest; Class 4: PF vs Rest.


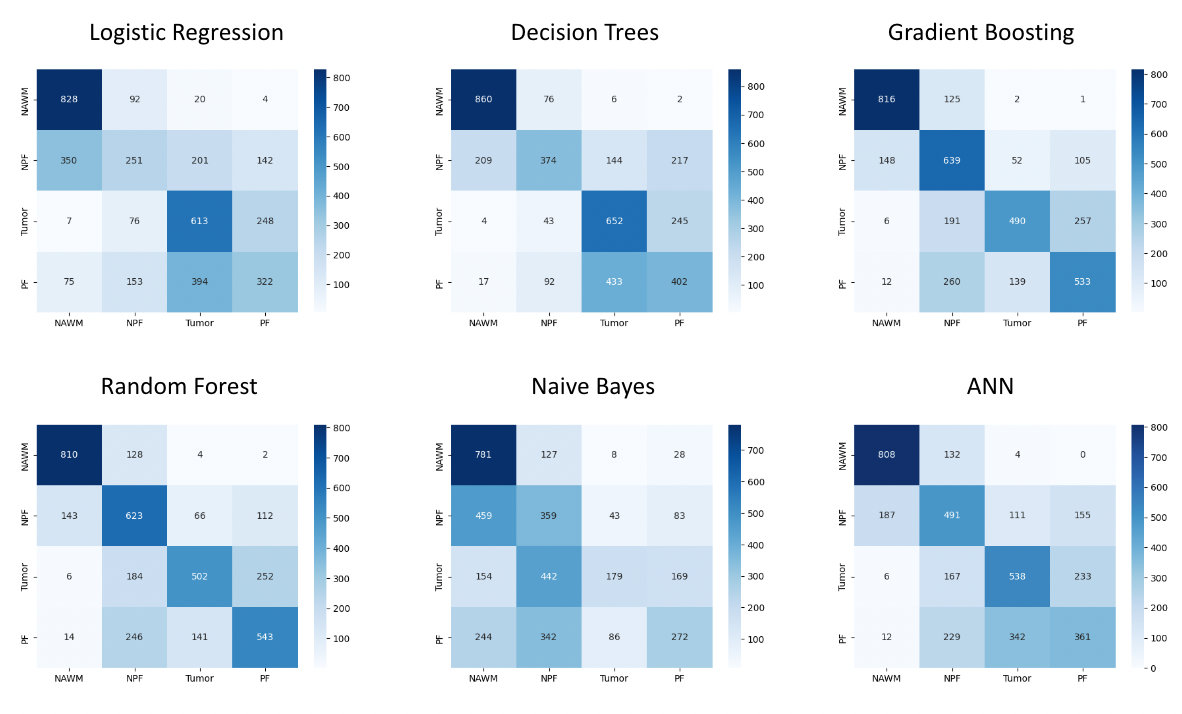


**Supplemental Figure 4:** Multiclass testing set confusion matrices. Y-axis represents machine-learning predictions. X-axis represents supervised classifiers.


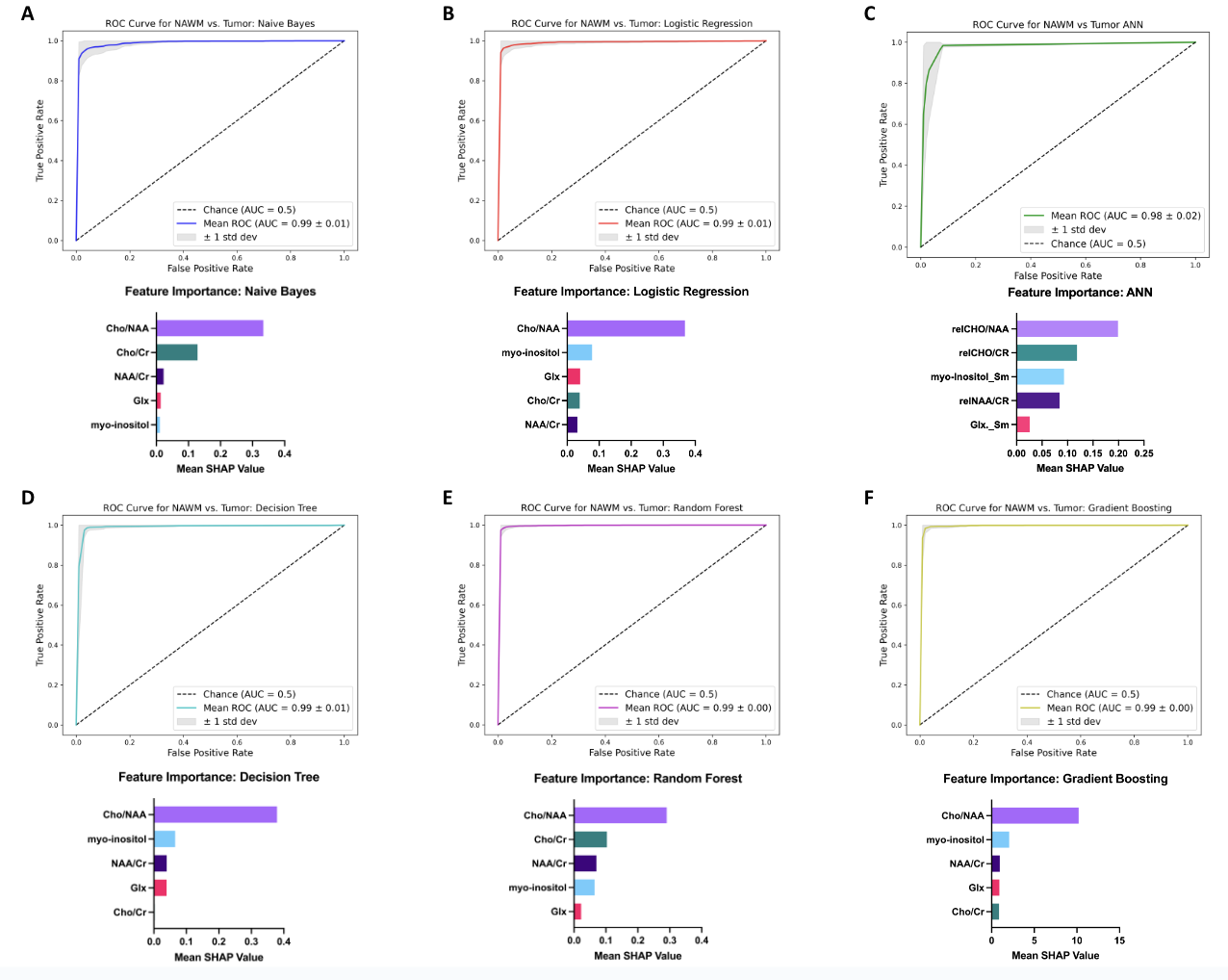


**Supplemental Figure 5:** NAWM vs. Tumor ROC plots and SHAP feature importance bar graphs.


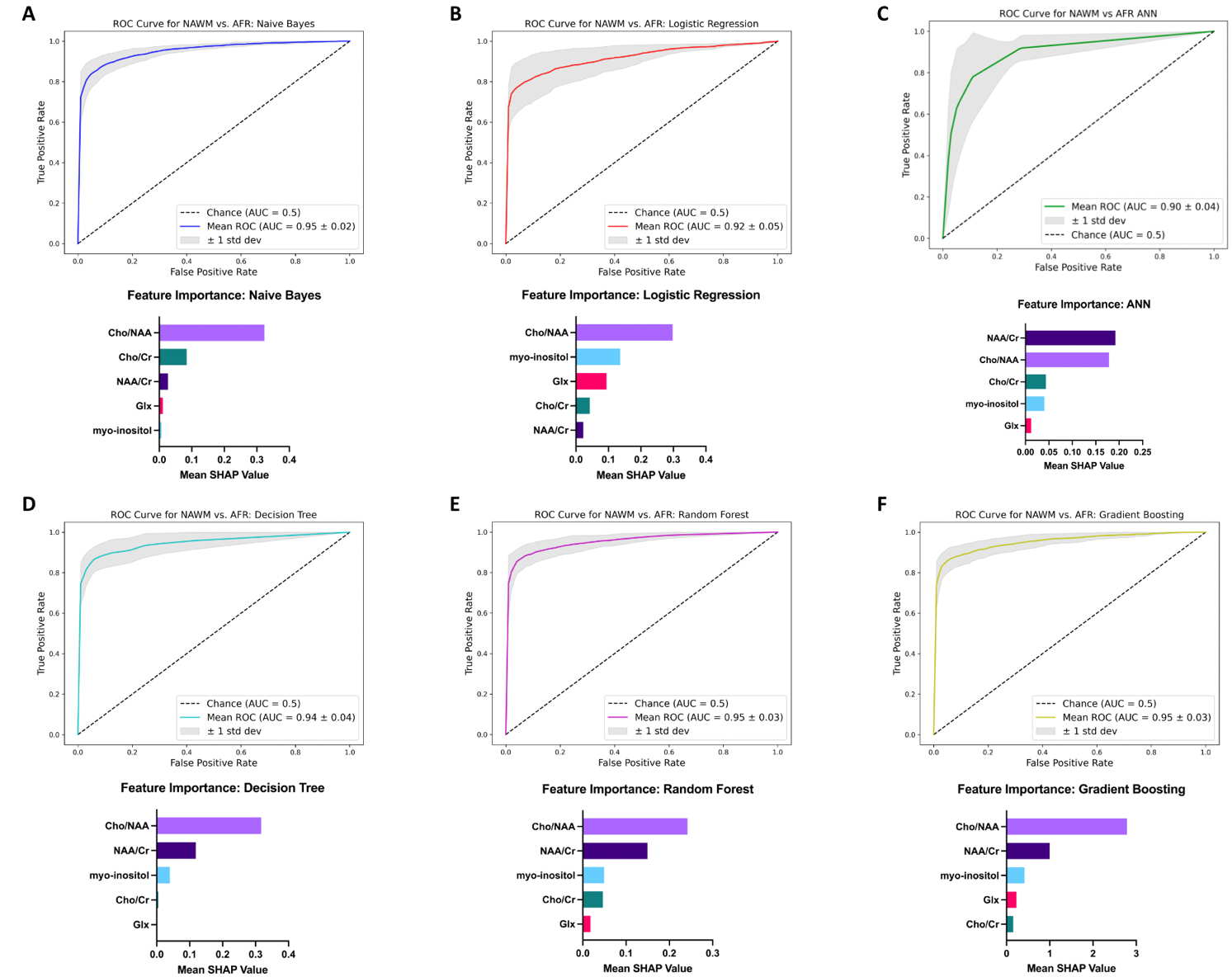


**Supplemental Figure 6:** NAWM vs. AFR ROC plots and SHAP feature importance bar graphs.


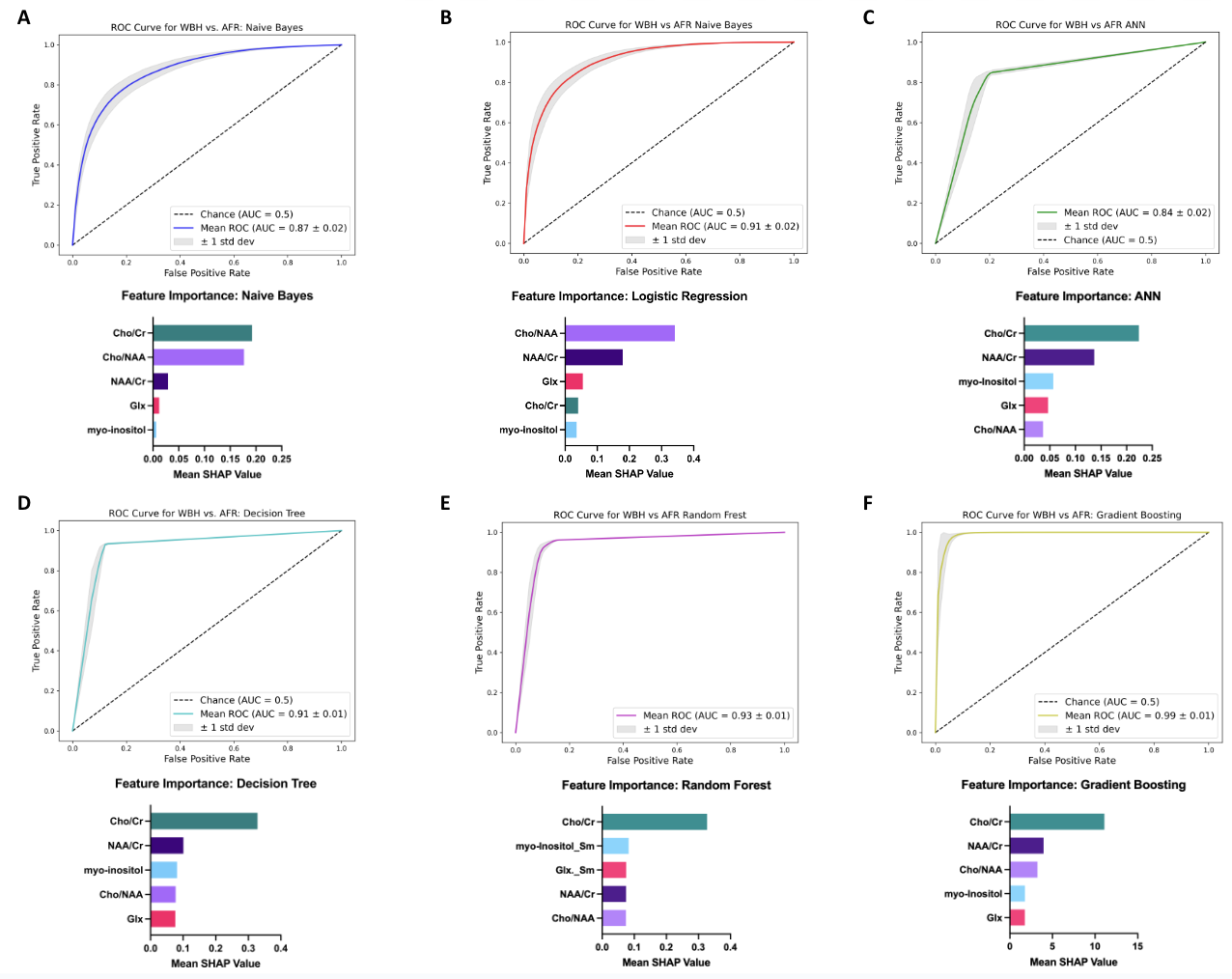


**Supplemental Figure 7:** WBH vs. AFR ROC plots and SHAP feature importance bar graphs.


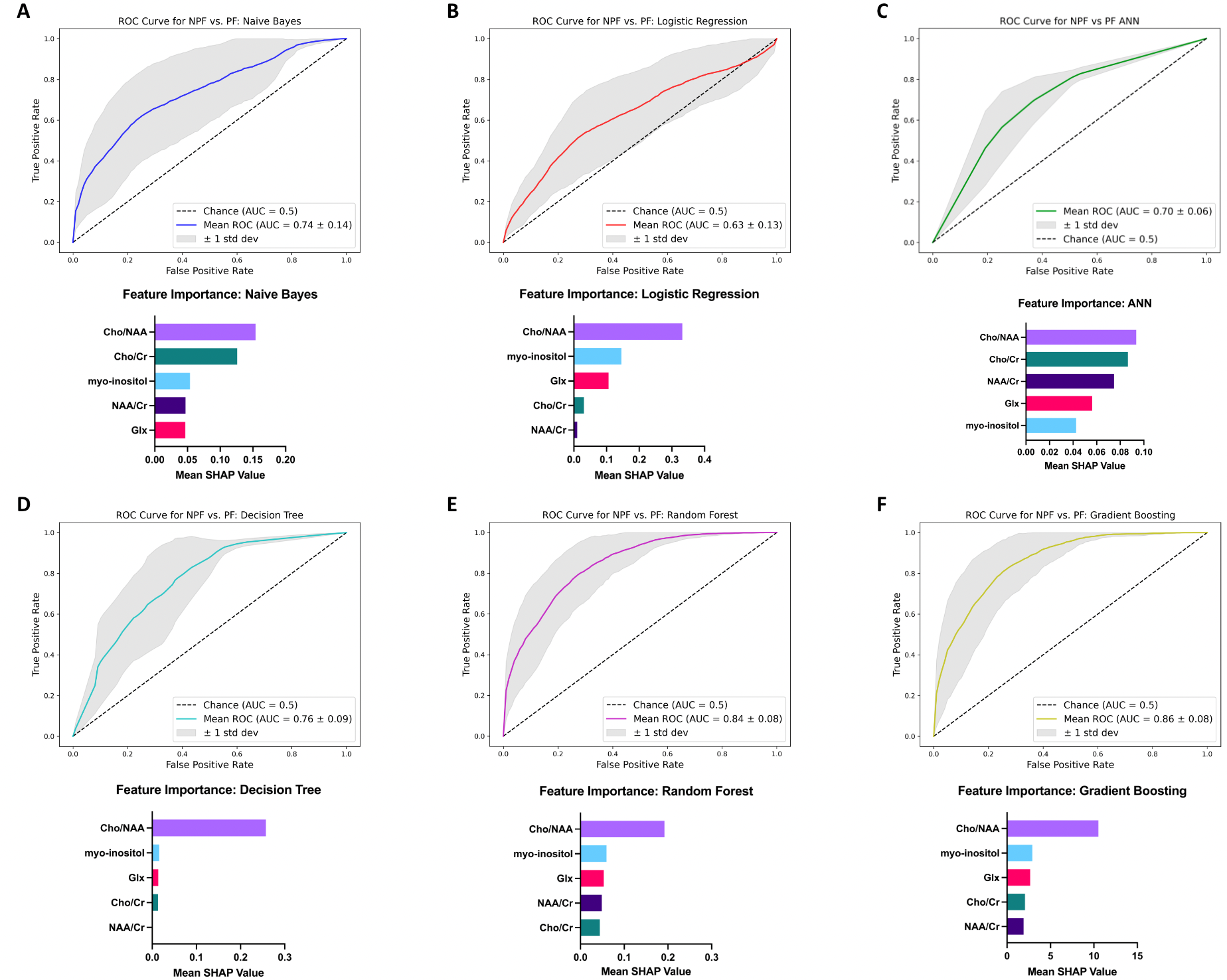


**Supplemental Figure 8:** NPF vs. PF ROC plots and SHAP feature importance bar graphs.


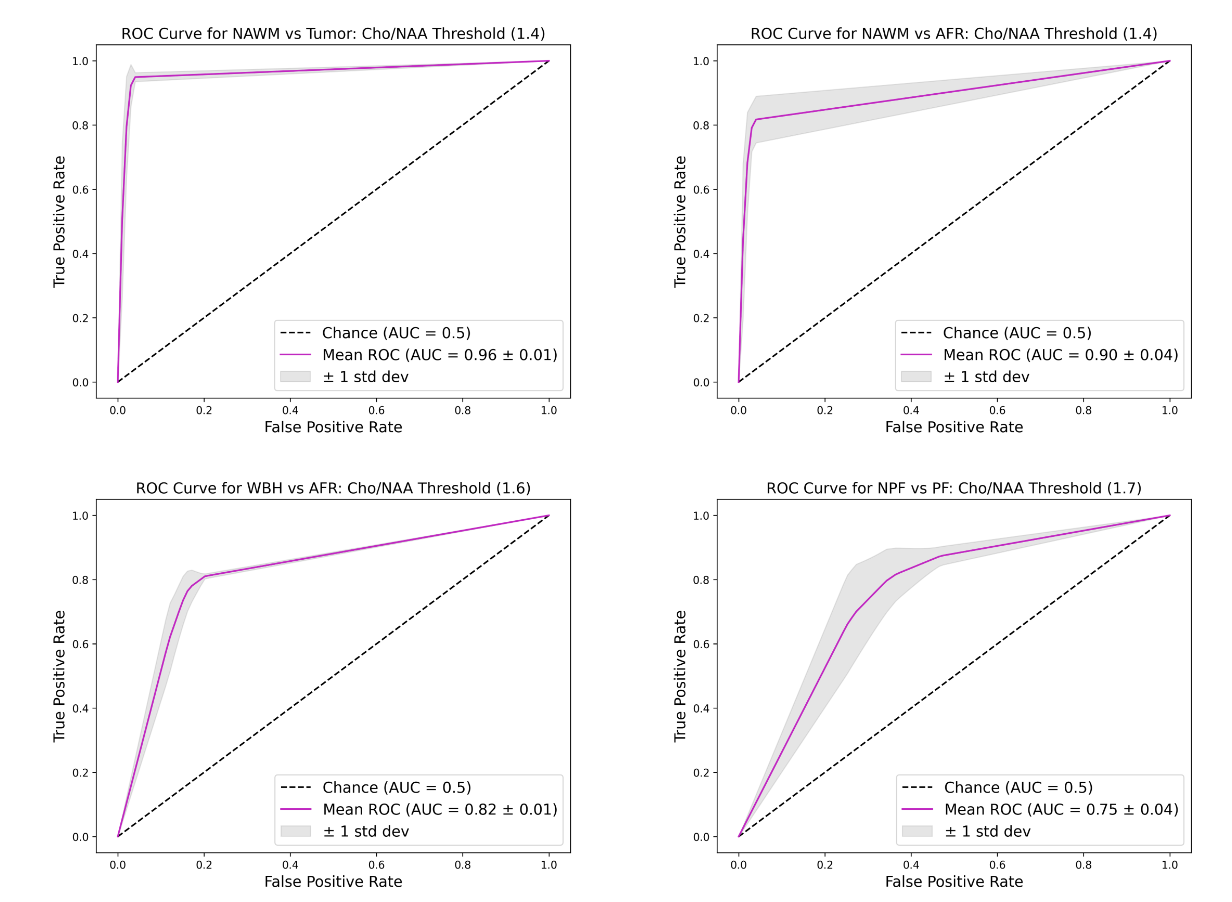


**Supplemental Figure 9:** Cho/NAA Threshold ROC plots with 5-fold cross-validation. Thresholds from 1.1 to 2.1 were tested with optimal mean AUC recorded. If peak mean AUC tied across groups, the threshold with the smallest standard deviation was selected. For NAWM vs AFR, a threshold value of 1.3 also produced a mean AUROC of 0.90 ± 0.04. For NPF vs PF, thresholds of 1.6 and 1.8 also produced the same mean AUROC of 0.75 ± 0.04.


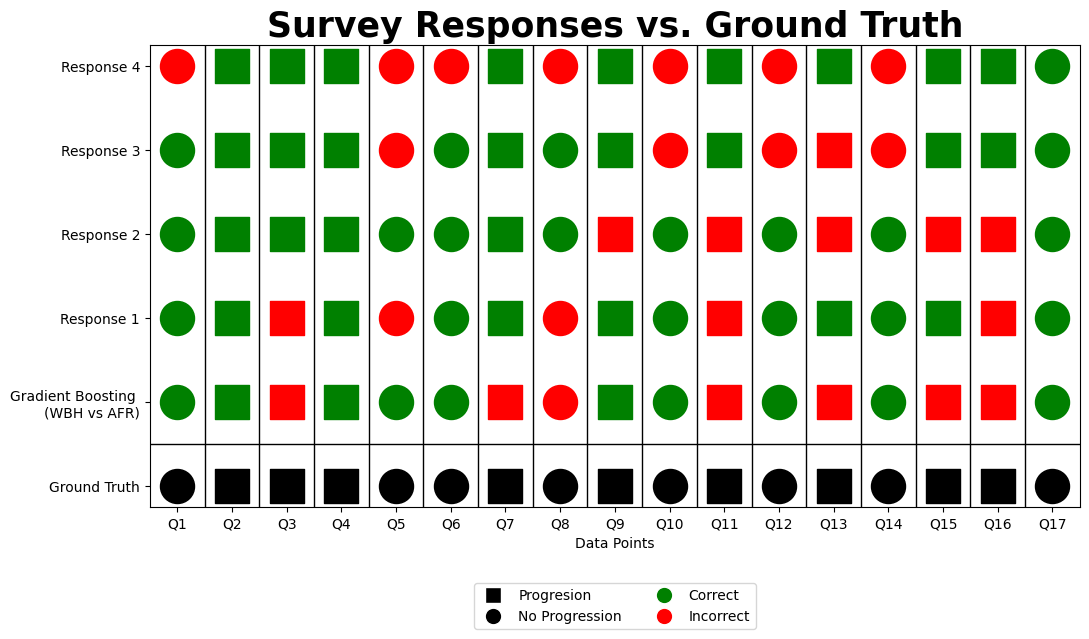


**Supplemental Figure 10**: Validation survey responses compared to Gradient Boosting (WBH vs AFR) and ground truth values. Cohen’s Kappa coefficient for the responses compared to the ground truth is 0.297, with the accuracy of the Gradient Boosting model of 70.6% for this subset of data.

**Supplemental Methods 1**

Imaging Protocol

MRI data was acquired using a 3 Tesla Siemens Skyra MRI Scanner with a 20-channel head/neck coil. WB-MRS data was acquired using a 3D echo planar spectroscopic imaging (EPSI) sequence.16,17 Imaging parameters: TR/TE/TI = 1550/17.6/198ms, excitation slab thickness = 140mm, FA = 710, FOV = 280x280x180mm, voxel resolution = 5.6x5.6x10, TA = 17min. Pre-contrast T1, T2 and FLAIR images and a post-contrast T1 image were also acquired.

WB-MRS data processing was done using the MIDAS. The metabolite maps were obtained using the FITT module and metabolite ratios were derived from these maps. The NAWM maps were derived from the T1 image segmentation white matter tissue maps from the contralateral side of the tumor. The FLAIR and enhancing ROIs were created by segmentation of the FLAIR image and subtraction of the pre-contrast T1 from the post-contrast T1 image, respectively. Metabolite data was extracted using automated spectral analysis from the MIDAS software for Cho, NAA, Cr, Glx, and myo-inositol. This data was pre-processed using a built-in quality map and linewidth filter between 2 and 12Hz.

Bone artifact around the temporal region has been shown to produce local magnetic field interference that decreases MR imaging quality^29^, resulting in a MIDAS quality score below acceptable threshold. With multifocal lesions, the MIDAS ROI auto-segmentation capabilities struggled to classify concurrently enhancing lesions, thus precluding analysis of multifocal neoplasms.

**Supplemental Methods 2**

Unsupervised Cluster Analysis

Voxel clusters were found by scaling voxels across all groups and conducting hierarchical clustering of all voxels in R using the *fastcluster* package. The associated clustering function was passed into the R package *pheatmap* to visualize voxel clusters across all 5 metabolite groups. Cluster statistics were extracted and summarized to display overall trends and quantity of voxels in each cluster **(Figure 4A)**.

**Supplemental Methods 3**

Hyperparameter Tuning

Hyperparameter tuning was performed using 10-fold cross validation randomized search with over 1000 iterations. This was implemented using the *GridSearchCV* and *RandomizedSearchCV* methods to achieve the best parameters with minimal error for each algorithm. The specific hyperparameters for each model are shown in detail in **Supplementary Table 4**.

**Supplemental Methods 4**

Feature Importance

Shapley Additive Explanation (SHAP) values were calculated to quantitatively interpret predictions and to determine feature importance. All SHAP values were computed using the *shap* package in Python.^19^

Model Performance

Model performance was evaluated primarily with an area under the receiver operating characteristic curve (AUC). Mean AUCs on the testing set with a 1 standard deviation were plotted following the same 5-fold cross validation used for model training. Multi-class AUC calculations used one-vs.-rest (OvR) methodology along with 5-fold cross validation, generating four values per model. Additional metrics such as the accuracy, precision, specificity, sensitivity, and F1-score were calculated on the testing set using the *scikit-learn.metrics* package in Python for binary models; these metrics were calculated from the confusion matrix OvR for multi-class models. All performance metrics are reported in **Supplementary Tables 1-3**.
